# Adalimumab improves health-related quality of life in patients with moderate to severe plaque psoriasis compared with the United States general population norms: Results from a randomized, controlled Phase III study

**DOI:** 10.1186/1477-7525-6-75

**Published:** 2008-10-02

**Authors:** Dennis A Revicki, Alan Menter, Steven Feldman, Miriam Kimel, Neesha Harnam, Mary K Willian

**Affiliations:** 1 Center for Health Outcomes Research, United BioSource Corporation, Bethesda, Maryland, USA; 2 Division of Dermatology, Baylor Research Institute, Dallas, TX, USA; 3 Department of Dermatology, Wake Forest University Baptist Medical Center, Winston-Salem, NC, USA; 4 Global Health Economics & Outcomes Research, Abbott Laboratories, Abbott Park, Illinois, USA

## Abstract

**Objective:**

To evaluate the impact of adalimumab on health-related quality of life (HRQOL) for patients with moderate to severe plaque psoriasis.

**Background:**

Psoriasis is a chronic, inflammatory, immune-mediated disease that has a significant impact on patients' HRQOL. Adalimumab is a fully human monoclonal antibody that blocks tumor necrosis factor, a pro-inflammatory cytokine, and is effective and well-tolerated for patients with moderate to severe psoriasis.

**Methods:**

Data were obtained for a secondary analysis of patients in a randomized, controlled Phase III trial evaluating the effect of adalimumab in patients with psoriasis (N = 1,205). Patients with moderate to severe psoriasis were randomized in a 2:1 ratio to adalimumab 80 mg (two 40 mg injections administered subcutaneously at baseline followed by one 40 mg injection every other week from Week 1 to Week 15) or placebo. Short Form-36 (SF-36) Health Survey scores of psoriasis patients were used to assess HRQOL and were compared with United States (US) population norms at baseline and Week 16.

**Results:**

Baseline Physical Component Summary (PCS) scores for the placebo and adalimumab groups were similar to the general US population. Baseline mean Mental Component Summary (MCS) scores were significantly lower for the adalimumab and placebo groups compared with the general population (47.4, 47.7, and 50.8 points, respectively; p < 0.0001). PCS scores at Week 16 for patients receiving adalimumab had improved and were significantly greater than scores for the general US population (52.7 vs 48.9; p < 0.001). Compared with the general US population, MCS scores at Week 16 were similar for patients receiving adalimumab (51.2 vs 50.8; p = 1.000) and lower for patients receiving placebo (50.8 vs 48.7; p < 0.0001).

**Conclusion:**

Psoriasis has a broad impact on patient functioning and well-being. Improvement in skin lesions and joint symptoms associated with adalimumab treatment was accompanied by improvements in HRQOL to levels that were similar to or greater than those of the general US population.

**Trial registration:**

Clinicaltrials.gov NCT00237887

## Introduction

Psoriasis is a chronic, inflammatory, immune-mediated disease that has significant impact on patients' health-related quality of life (HRQOL) [1-7]. Psoriasis symptomatology, including pain and itching, combined with concerns about the appearance of one's skin can substantially affect a patient's psychological well-being and can result in emotional distress, a sense of stigmatization, worry, and embarrassment. Deficits in social and sexual functioning, as well as social, recreational, and work activity restrictions have all been reported in patients with psoriasis. A survey of National Psoriasis Foundation members with severe psoriasis found that the disease negatively impacted the HRQOL of nearly 80% of respondents [8]. HRQOL outcomes provide greater insight into the impact of psoriasis on patient functioning and well-being than do clinical measures, such as the percentage of body surface area (BSA) affected by psoriasis [9].

To more fully understand the impact that psoriasis and its treatments have on a patient's functioning and well-being, it is important that clinical trials of new psoriasis treatments assess patient HRQOL. Successful treatment of moderate to severe psoriasis with TNF antagonists improves physical function, as well as social and psychological aspects of psoriasis [10-17]. Adalimumab, a fully human monoclonal antibody that blocks TNF, is effective and well-tolerated for patients with moderate to severe psoriasis [16-19].

In a 16-week, Phase III, randomized, double-blind, placebo-controlled trial, adalimumab improved HRQOL outcomes in patients with psoriasis, as measured with both the Dermatology Life Quality Index (DLQI) and the Mental and Physical Component Summary scores of the Short Form-36 Health Survey (SF-36) [16,17]. However, the meaning associated with the magnitude of change in HRQOL scores is not well-characterized. There is currently little guidance for interpreting changes in HRQOL scores. Most often information on minimum clinically important differences for HRQOL scores are determined by anchor-based and distribution-based methods [20,21]. Relevant anchors may be clinical endpoints, global clinician or patient ratings of improvement in symptom or health status, or other measures. It is important for clinicians to understand that changes in HRQOL scores also reflect changes in clinical status or function and may therefore impact treatment decisions. Criterion-based interpretation, which uses the relationship of these scores to external variables or population norms to assign meaning, is one approach to relating the importance of scores in terms that are more easily understood by clinicians and patients [2].

The objective of this secondary analysis was to evaluate the effect of adalimumab on initial improvement in HRQOL for patients with psoriasis compared with the general population of the United States (US). The norm-based approach compared mean HRQOL scores in a target patient group (in this case psoriasis) to age-, sex-, and race-matched mean HRQOL scores from members of the general US population. This information then allowed the quantification of HRQOL burden before and after treatment, and also demonstrated improvements in health outcomes based on comparisons between the clinical study patients and the general US population, adjusted for age, sex, and race.

## Patients and methods

### Patient population

#### Moderate to severe psoriasis patient sample

The data for this secondary analysis came from the **R**andomized Controlled **EV**aluation of Adalimumab **E**very Other Week Dosing in Moderate to Severe Psoriasis Tri**AL **(REVEAL) study, a 52-week, Phase III clinical trial in adult patients with moderate to severe chronic plaque psoriasis [23]. Patients were randomized in a 1:2 ratio to receive subcutaneous injections of placebo only or adalimumab 80 mg at Week 0 followed by 40 mg every other week from Week 1 to Week 15 during the initial 16-week, double-blind treatment period. Following the initial treatment period, all patients who achieved at least 75% improvement in Psoriasis Area and Severity Index (PASI) scores (PASI 75 response) received adalimumab 40 mg every other week. Because few placebo-treated patients (17.3%; n = 57) were observed after Week 16 and all patients received adalimumab after Week 16, the analyses reported here are based only on data collected at baseline and at Week 16.

To be eligible for the study, patients were required to meet the following disease severity indices at the baseline visit: moderate to severe plaque psoriasis defined as ≥ 10% BSA involvement, a PASI score of ≥ 12 and a Physician's Global Assessment of at least moderate disease. The REVEAL study was conducted in accordance with the principles of the Declaration of Helsinki, and all study sites received approval from independent ethics committees. All patients provided written, informed consent before any study-related procedures were performed.

#### US general population samples

General population normative data came from two sources: the 1998 National Survey of Functional Health Status (NSFHS) and the 2002 Medical Expenditures Panel Survey (MEPS). The NSFHS is a representative sample (N = 1,982) of the non-institutionalized adult population in the United States [24,25] and was included to allow normative comparisons for SF-36 Health Survey (Version 1) scale scores between the general US population and REVEAL study patients. The MEPS is a nationally representative, cross-sectional sample of the non-institutionalized US civilian population (N = 23,517) [26]. To use more recent data, MEPS health status data were employed. This survey also enabled similar comparisons for the SF-36 Physical Component Summary (PCS) and Mental Component Summary (MCS) scores and allowed adjustment for age, sex, and race in the data analyses.

### Instruments used for assessment of health-related quality of life

The SF-36 Health Survey (Version 1) is a 36-item general health status instrument often used in clinical trials and health services research. The SF-36 consists of 8 scales: Physical Function, Role Limitations-Physical, Vitality, General Health Perceptions, Bodily Pain, Social Function, Role Limitations-Emotional, and Mental Health [7]. Two overall summary scores (PCS and MCS) are obtained from the SF-36. Norm-based scoring algorithms were used for the scales and for the PCS and MCS scores, which are normed to a mean of 50 and standard deviation of 10, with greater scores indicating better health. Change scores of 2 to 3 points for the individual normed scales, equivalent to a 0.2 to 0.3 effect size, can be used as guidelines to interpret clinically meaningful differences; differences of 2 to 3 points for the summary scores are considered the minimum important difference in the general US population [7]. There is extensive evidence demonstrating the reliability and validity of the SF-36 [4] in general populations. The SF-36 has also demonstrated acceptable reliability, validity, and responsiveness to change in dermatology populations [9]. The SF-36 was included in both the REVEAL and NSFHS studies.

The Short Form-12 Health Survey (SF-12) (Version 1), which was used in the MEPS survey included in this analysis, contains 12 items from the SF-36 Health Survey, with 1 or 2 items measuring each of the 8 concepts included in the SF-36. SF-12 summary scores (PCS and MCS) are normed with a general population mean of 50 and standard deviation of 10, and greater scores reflect better health status. The psychometric properties of the SF-12 are well-established in various diseases [25]. Instrument developers have demonstrated that SF-12 and SF-36 summary scores are comparable [25].

### Statistical analyses

#### Descriptive statistics

Descriptive statistics were reported for demographic and clinical characteristics of the REVEAL sample. Means and standard deviations were used to describe continuous variables, whereas frequency distributions were used to describe categorical variables. Student *t*-tests were used to compare independent groups and chi-square tests were used to compare score differences between groups. The sample for the current analyses was comparable to that of the HRQOL analysis for the REVEAL trial [30] and included all randomized patients who had completed the baseline primary HRQOL assessment (based on the DLQI and SF-36) and at least one follow-up assessment within 16 weeks of study entry.

#### Assessing the burden of psoriasis on health-related quality of life in psoriasis

SF-36 scale score norms were derived from NSFHS data collected in 1998 [24] for men and women 45 to 50 years of age (similar to the REVEAL sample) and were compared with baseline SF-36 scale scores for each of the REVEAL treatment groups (adalimumab and placebo) before treatment initiation. PCS and MCS scores for each of the REVEAL treatment groups were also compared with US normative data derived from the more recent MEPS survey.

#### Assessing the impact of treatment on health-related quality of life in psoriasis

SF-36 scale score norms for men and women 45 to 54 years of age from the NSFHS were compared with SF-36 scale scores by treatment group at Week 16 [24]. To compare PCS and MCS scores from the REVEAL study sample to the MEPS data sample (US general population norms), two analyses were performed. First, separate least squares regression models for PCS and MCS scores from the REVEAL study were compared with summary scores from MEPS adjusted for age, sex, and race. The F-test was used to test for the group factor and the Bonferroni method was used to adjust for multiple comparisons. Second, a matched-case analysis was performed. For each patient in the REVEAL study, 5 age-, sex-, and race-matched controls were randomly chosen from the MEPS data, except in cases for which fewer than 5 controls were available. Student *t*-tests for independent groups were used to assess mean score differences between groups. PCS and MCS scores for the REVEAL study were calculated from the SF-36, whereas the PCS and MCS scores for the MEPS were calculated from the SF-12.

## Results

### Demographics and clinical characteristics

A total of 1,205 patients from the REVEAL study were included in this analysis: 808 patients received adalimumab and 397 patients received placebo. Baseline demographic and clinical characteristics were similar between the two treatment groups and were indicative of moderate to severe plaque psoriasis (Table 1).

**Table 1:** Baseline characteristics for all eligible patients in the REVEAL study

	**Treatment Group**		
		
	**Adalimumab (N = 808)**	**Placebo (N = 397)**	**P-value^1^**
**Age (yrs)**, mean ± SD^1^	44.1 ± 13.2	45.4 ± 13.4	0.1177
**Sex**, n (% female)^1^	266 (32.9)	141 (35.5)	0.3996
**Race**, n (%)^2^			0.5432
White	738 (91.3)	358 (90.2)	
Black	27 (3.3)	20 (5.0)	
Asian	21 (2.6)	7 (1.8)	
American Indian/Alaska Native	3 (0.4)	1 (0.3)	
Other	19 (2.4)	11 (2.8)	

**Ethnicity**, n (%)^2^			0.3434
Not Hispanic or Latino	754 (93.3)	364 (91.7)	
Hispanic or Latino	54 (6.7)	33 (8.3)	

**Psoriasis history**			
Duration of psoriasis (yrs), mean ± SD^1^	18.6 ± 12.0	18.8 ± 12.0	0.7309
Concomitant psoriatic arthritis, (% yes)^2^	222 (27.5)	113 (28.5)	0.7326
Prior systemic psoriasis therapy, (% yes)^2^	260 (32.2)	128 (32.2)	1.0000

**Psoriasis baseline assessments**			
Percentage of body surface area affected by psoriasis, mean ± SD^2^	25.8 ± 15.5	25.6 ± 14.8	0.8796
Psoriasis Area and Severity Index, mean ± SD^2^	19.0 ± 7.1	18.8 ± 7.1	0.7787

**Physician's global assessment score, n (% yes)^2^**			1.0000
Moderate^2^	416 (51.5)	219 (55.2)	
Severe	341 (42.2)	155 (39.0)	
Very severe	51 (6.3)	23 (5.8)	

^1^*t*-Test.

^2^Fisher exact test or chi-square test.

^3^P-value for comparison between adalimumab versus placebo.

### Assessing the burden of psoriasis on health-related quality of life

#### SF-36 summary scores

Based on the 2002 MEPS data, baseline PCS scores for patients in the REVEAL study were similar to the general US population for those receiving adalimumab (adalimumab mean = 48.9 vs MEPS mean = 48.9; p = 0.2636) and placebo (placebo mean = 49.1 vs MEPS mean = 48.9; p = 1.000). Mean MCS scores were significantly lower for the adalimumab and placebo treatment groups compared with the MEPS sample (47.4, 47.7, and 50.8 points, respectively; p < 0.0001 for both treatment groups). Similar findings were observed for the matched-case analysis.

#### SF-36 scale scores

Table 2 summarizes the baseline SF-36 scale scores for the REVEAL treatment groups and for the general US population based on men and women 45 to 54 years of age in the NSFHS study. Patients in each REVEAL treatment group generally had lower baseline SF-36 scale scores compared with the general US population, with the largest differences seen in Social Function (-4.05 and -3.96 points) and Role-Emotional (-3.00 and -3.20 points) scores for the adalimumab and placebo groups, compared with the general US population. These observed differences are considered clinically meaningful, given they exceed the 3.0 point minimum clinically important difference (MCID) criteria.

**Table 2:** HRQL impact of psoriasis at baseline: study population versus general US population

	**Adalimumab Mean (SD)^1^**	**Placebo Mean (SD)^2^**	**General US Population^3 ^Mean (SD)**
Physical Functioning	48.6 (10.3)	48.2(10.6)	49.4 (10.0)
Role-Physical	48.2 (11.1)	48.7 (10.8)	50.1 (9.9)
Bodily Pain	47.5 (10.9)	47.4 (10.6)	49.2 (10.2)
General Health	49.5 (9.5)	50.2 (9.5)	49.3 (10.7)
Vitality	49.9 (9.8)	50.3 (10.3)	50.4 (10.5)
Social Functioning	46.0 (11.8)	46.1 (11.7)	50.1 (10.1)
Role-Emotional	47.6 (12.0)	47.4 (12.0)	50.6 (9.5)
Mental Health	47.4 (10.8)	47.9 (11.0)	49.4 (10.7)

^1^N = 804–808.

^2^N = 396–397.

^3^SF-36 values for males and females aged 45 to 54 years from SF-36 manual (1998 population); N = 417.

### Assessing the impact of treatment on health-related quality of life in psoriasis

#### SF-36 summary scores

**Figure 1:**
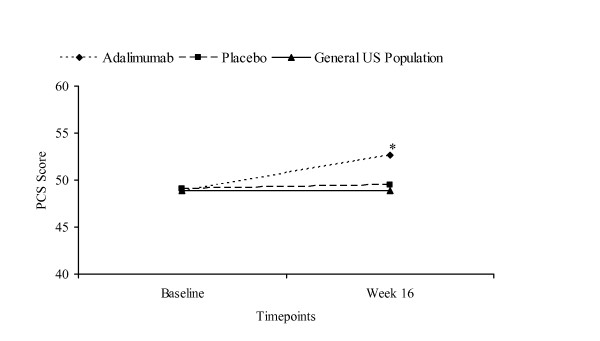
**Mean PCS Scores Across 16 Weeks for REVEAL Trial Groups Versus General US Population**. General US population is the entire MEPS population (N = 23,517). SF-12 values from entire MEPS population controlled for age, sex, and race. Sample size for adalimumab group at baseline and Week 16: n = 805 and n = 755. Sample size for placebo group at baseline and Week 16: n = 396 and n = 354. Analysis of covariance with Bonferroni adjustment for multiple comparisons. *p < 0.0001

**Figure 2:**
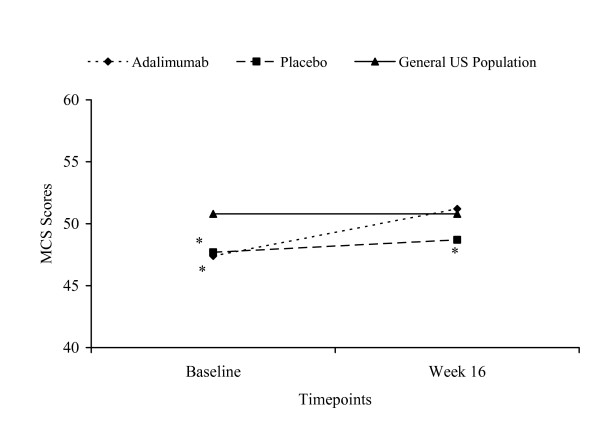
**Mean MCS Scores Across 16 Weeks for REVEAL Trial Groups Versus General US Population**. General US population is the entire MEPS Population (N = 23,517). SF-12 values from entire MEPS population controlled for age, sex, and race. Sample size for adalimumab group at baseline and Week 16: n = 805 and n = 775. Sample size for placebo group at baseline and Week 16: n = 396 and n = 354. Analysis of covariance with Bonferroni adjustment for multiple comparisons. *p < 0.001, **p < 0.0001.

Using age-, sex-, and race-adjusted data from the 2002 MEPS sample, patients receiving adalimumab were observed to have significantly greater mean PCS scores at Week 16 compared with those of the general US population (adalimumab mean = 52.7 vs MEPS mean = 48.9; p < 0.001) (Figure 1). The MCS scores at Week 16 were similar between those receiving adalimumab and the general population (adalimumab mean = 51.2 vs MEPS mean = 50.8; p = 1.000) while patients receiving placebo had lower scores when compared with the general US population (placebo mean = 48.7 vs MEPS mean = 50.8; p < 0.0001) (Figure 2).

Similar findings were observed in comparing PCS and MCS scores of the REVEAL treatment groups with the general population based on the age-, sex-, and race-matched 2002 MEPS data (data not shown). At Week 16, mean PCS scores for those receiving adalimumab were significantly greater than mean scores for the general US population (adalimumab mean = 52.7 vs MEPS mean = 49.3; p < 0.0001) and MCS scores were similar to those for the general US population (adalimumab mean = 51.2 vs MEPS mean = 50.3; p = 0.2440). After 16 weeks, the mean PCS scores of placebo-treated patients were similar to those for the general US population (placebo mean = 49.5 vs MEPS mean = 49.3; p = 0.8052) while the mean MCS scores were lower than those for the general US population (placebo mean = 48.7 vs MEPS mean = 50.3; p = 0.0005).

#### SF-36 scale scores

At Week 16, mean scores for all SF-36 scales had improved for patients receiving adalimumab therapy and were similar to or greater than scores for the NSFHS sample (Table 3). The largest score improvements (baseline to Week 16) were seen for Bodily Pain and Social Function (+6.8 and +5.3 points, respectively), while the largest differences in scores between the adalimumab group and the general US population were seen for Bodily Pain, Vitality, and General Health (+5.1, +2.8, and +2.5 points, respectively, in favor of adalimumab). The differences seen in Bodily Pain are clinically significant (ie, exceeding 3.0 points). For those receiving placebo, mean scores for all SF-36 scales at Week 16 were similar to those seen at baseline and were lower – except for General Health and Vitality – compared with the NSFHS sample (range -2.3 to +0.9).

**Table 3:** HRQL impact of psoriasis at Week 16: study population versus general US population

	**Adalimumab Mean (SD)^1^**	**Placebo Mean (SD)^2^**	**General US Population^3 ^Mean (SD)**
Physical Functioning	51.3(9.0)	48.6 (10. 4)	49.4 (10.0)
Role-Physical	52.3(8.4)	49.4 (10.6)	50.1 (9.9)
Bodily Pain	54.3(9.3)	48.9 (10.9)	49.2 (10.2)
General Health	51.8(8.6)	50.2 (9.3)	49.3 (10.7)
Vitality	53.2(9.2)	50.5 (10.4)	50.4 (10.5)
Social Functioning	51.4(8.5)	47.2 (10.9)	50.1 (10.1)
Role-Emotional	51.3(8.9)	48.3 (11.8)	50.6 (9.5)
Mental Health	51.1(9.2)	49.1 (11.0)	49.4 (10.7)

^1^N = 774–775.

^2^N = 354.

^3^SF-36 values for males and females aged 45 to 54 years from SF-36 manual (1998 population); N = 417.

## Discussion

This study measured the burden of psoriasis on patient functioning and well-being based on a baseline comparison between patients with moderate to severe psoriasis from the REVEAL study and two US general population samples. Regardless of the data source defining the general US population, patients with psoriasis reported similar physical functioning and more impaired mental health functioning compared with general population norms, even after adjusting for age, sex, and race. These findings differ from those observed based on SF-36 summary scores in a previous psoriasis clinic sample [5]; however, the earlier study did not control for differences in age, sex, or ethnicity in the data analyses.

Based on SF-36 summary scores, there was a significant mental health burden observed in patients with moderate to severe psoriasis. The difference in MCS scores between the REVEAL and the MEPS samples were 3.1 to 3.4 points, which exceeded the MCID of 3.0 points. This finding is not surprising given that previous studies have indicated that patients with psoriasis are more likely to report depressive symptoms [31,32]. Although the summary scores for the physical components of health at baseline were similar between both treatment groups and the general US population, SF-36 scale scores indicate that aspects of physical health such as bodily pain are significantly impaired by psoriasis. In a previous study, Rapp *et al*. also demonstrated differences in Bodily Pain, Physical Functioning, and other SF-36 scale scores between a psoriasis sample and the NSFHS sample [5].

There were no evident baseline differences in physical health summary outcomes between REVEAL study groups and the general US population; however this finding may not be due to the nature of disease impact on physical aspects of health, but rather to the method used to derive PCS scores. PCS scores are based on the positive coefficients of physical health-related scales (ie, Physical Function, Bodily Pain, etc.) and negative coefficients of mental health-related scales (ie, Mental Health, Vitality, etc.). Previous research has demonstrated that the SF-36 summary scores may be less informative in situations where there is impact on both physical and emotional functioning [33,34]. However, based on the NSFHS data only minor differences were seen between the REVEAL and general populations groups on physical functioning and bodily pain, but there were clinically meaningful differences for social functioning and role-emotional scores.

We observed significant improvements in MCS and PCS scores after 16 weeks of adalimumab treatment compared with US population norms, while the placebo groups demonstrated stability in scores over the treatment course. The finding that both emotional and physical health were influenced by treatment is consistent with findings observed in three other psoriasis trials that employed the SF-36 [14,15,18]. Although all three studies demonstrated significant improvements in all SF-36 scales, the greatest baseline to endpoint improvements (range of trial durations: 10 to 16 weeks) were seen in the Bodily Pain and Social Function scales. These findings are also consistent with those reported for infliximab after 10 weeks of treatment, where Physical Function, Bodily Pain, General Health, Vitality, Social Function, and Mental Health scale scores were greater than or similar to population norms [14].

There were several limitations associated with the normative comparisons and our analyses. First, the current analysis was based only on SF-36 normative data in the United States. However, patients with psoriasis participating in the REVEAL study were recruited from the United States and Canada. It is possible that our findings may be affected by differences in normative scores across different countries. Second, the health status scores were based on patient self-reports and may be associated with some bias in reporting. However, this bias may be minimal given the strong associations between the clinical endpoints and the HRQOL measures in this study [14]. Third, the SF-36 summary scores may sometimes yield aberrant results which are partially explained by the negative weight for Mental Health and Vitality in scoring the PCS. Finally, longer term data will be beneficial in confirming the results of this study.

## Conclusion

This analysis confirms that psoriasis has a broad impact on patients' HRQOL. Adalimumab treatment of patients with psoriasis improved the physical and psychological health of these patients to levels comparable with or greater than the physical and psychological health of the general population in the United States.

## List of abbreviations

BSA: Body Surface Area; DLQI: Dermatology Life Quality Index; HRQOL: Health-Related Quality of Life; MEPS: Medical Expenditures Panel Survey; MCS: Mental Component Summary; NSFHS: National Survey of Functional Health Status; PASI: Psoriasis Area and Severity Index; PCS: Physical Component Score; REVEAL: Randomized Controlled Evaluation of Adalimumab Every Other Week Dosing in Moderate to Severe Psoriasis Trial; SF-12: Short Form-12 Health Survey; SF-36: Short Form-36 Health Survey

## Competing interests

DAR: Dr Revicki is an employee of United BioSource Corporation and completed this analysis on behalf of Abbott Laboratories. AM: Dr. Menter has received research support and/or lecture honoraria from Abbott, Amgen, Astellas, Biogen, Centocor, Genentech, and Wyeth. MK: Dr Kimel is an employee of United BioSource Corporation and completed this analysis on behalf of Abbott Laboratories. NHH: Ms Harnan is an employee of United BioSource Corporation and completed this analysis on behalf of Abbott Laboratories. MKW: Dr Willian was an employee of Abbott Laboratories at the time the analysis was completed. SF: Dr. Feldman owns stock or stock options in Medical Quality Enhancement Company and Photomedex. He has received grants from Abbott, Galderma, Astellas, and Warner Chilcott and has received honoraria from Abbott, Centocor, Genentech, Amgen, Warner Chilcott, Astellas, Suncare, Galderma, Stiefel, Doak, and Novartis.

## Authors' contributions

DAR, MK, NH, and MKW designed and interpreted the analysis of health-related quality of life data. AM and SF assisted Abbott Laboratories with the original Phase III study design and acquisition of data. All authors were involved in drafting the manuscript and critically reviewing it for intellectual content. All authors approved the final version of the manuscript for publication.
